# Dexmedetomidine in neonates: utilisation trends and safety profile over time in a neonatal intensive care unit

**DOI:** 10.1136/bmjpo-2024-003004

**Published:** 2025-03-18

**Authors:** Gozdem Kayki, Nadir Yalcin, Hasan Tolga Celik, Sule Yigit

**Affiliations:** 1 Department of Pediatrics, Division of Neonatology, Hacettepe University Faculty of Medicine, Ankara, Turkey; 2 Department of Clinical Pharmacy, Hacettepe University, Faculty of Pharmacy, Ankara, Turkey; 3 KU Leuven, Leuven, Belgium

**Keywords:** Infant, Analgesia, Neonatology

## Abstract

**Background:**

Dexmedetomidine is an alpha-2 adrenergic agonist with sedative, anxiolytic and analgesic effects. Its use in neonatal intensive care units (NICUs) has been increasing in the last decade. The aim of this study was to assess the safety profile of dexmedetomidine and to identify specific trends in its use over time.

**Methods:**

In this retrospective observational study, data were collected on all patients who received continuous infusion of dexmedetomidine in a level IV NICU in Turkey between 2018 and 2023. Demographic characteristics were compared between preterm and term infants using the Mann-Whitney U test. Differences in adverse effects between term and preterm infants, as well as between lower and higher doses, were analysed using the χ^2^ test. Regression analysis was conducted to identify factors influencing adverse effects.

**Results:**

A total of 383 patients were included. The participants had a median (IQR) gestational age of 37 (35–38) weeks with a median (IQR) birth weight of 2700 (2140–3270) grams and the median (IQR) postmenstrual age at the time of dexmedetomidine initiation was 38 (36–40) weeks. The most common indication for use was pain control following surgery and/or interventional procedures (81.5%). There was a statistically significant increase in initial doses (p<0.001) and treatment duration (p=0.009). Adverse drug reactions (ADRs) were observed in 5% of cases, mostly bradycardia (50%) and ADRs did not correlate with the dose (0.80), treatment duration (0.96) or gestational age (p=0.93).

**Conclusion:**

Our data and experiences demonstrated a significant increase in the dose and duration of dexmedetomidine use in the NICU over the years. Additionally, the findings have suggested that higher doses and treatment duration do not result in an increase in ADRs during the acute period.

**WHAT IS ALREADY KNOWN ON THIS TOPIC**

- Dexmedetomidine has gained popularity and use in neonatal intensive care units (NICUs) has notably increased in recent years, with reports indicating a 50-fold surge over the past decade.

**WHAT THIS STUDY ADDS**

- This study has shown that the dose and duration of dexmedetomidine with a safe profile in the NICU have increased significantly over the years.

**HOW THIS STUDY MIGHT AFFECT RESEARCH, PRACTICE OR POLICY**

- The findings suggest that the initial dosing may have been insufficient, necessitating higher doses to achieve the desired therapeutic effect. This observation underscores the need to re-evaluate dosing strategies for dexmedetomidine to ensure optimal efficacy.

## Introduction

Pain in the neonatal period can lead to permanent neuroanatomical irregularities, altered brain maturation, emotional and behavioural challenges, and learning disabilities.^1 2^ Therefore, early recognition and management during this critical period is important.^3^ In the neonatal period, benzodiazepines and opioids are commonly used for sedation and analgesia.^4^ However, these medications pose risks such as tolerance development, withdrawal, respiratory depression, and poor neurodevelopmental outcomes in extremely preterm infants.^5 6^

Dexmedetomidine has been considered a promising option for neonatal sedation and analgesia because of its minimal depressant effects on the respiratory and gastrointestinal systems.^7^,^9^ Additionally, it is thought to have some potential neuroprotective effects. Since experimental studies have demonstrated its anti-apoptotic effects across various models of brain injury.^10 11^ Therefore, it has gained popularity in recent years and its use in neonatal intensive care units (NICUs) has increased notably, with reports of a 50-fold increase over the last decade.^12 13^ This has also led to changes in our practice, and dexmedetomidine has become the preferred choice for pain management in our NICU since 2018.

Dexmedetomidine is a selective alpha-2 adrenergic receptor agonist.^14^,^16^ Its analgesic effects are attributed to the activation of the receptors in the dorsal horn of the spinal cord, which reduces the release of substance P. Additionally, its sedative and anxiolytic effects originate in the locus coeruleus of the brainstem, where stimulation of alpha-2 receptors activates inhibitory neurons. In early efficacy studies, dexmedetomidine was reported to be well-tolerated in both preterm and term infants, with no serious adverse drug reactions (ADRs) detected.^4^ It has been shown to improve comfort scales in critically ill neonates.^4 17^ Additionally, its use has also been documented in other special patient groups, such as postoperative patients and neonates with encephalopathy undergoing therapeutic hypothermia.^15 18^ Hypotension and bradycardia are the common documented potential ADRs associated with dexmedetomidine. However, previous studies did not observe these ADRs leading to severe clinical impact.^8^

Therefore, we wanted to conduct a comprehensive evaluation of our experience with the use of dexmedetomidine. We hypothesise that this drug has a favourable safety profile and that its use patterns have evolved due to increased clinical experience. The objectives of the study were to assess the safety profile of dexmedetomidine, to identify factors influencing ADRs and to analyse specific usage trends over the study period.

## Material and methods

### Study design

This study was designed as a retrospective observational study involving a chart review of patient records from 2018 to 2023. It was conducted in a level IV NICU at a referral hospital in Ankara, Türkiye, specialising in the care of neonates with surgical and cardiac conditions.

### Patient involvement

Patients who were admitted to the NICU during the study period and received dexmedetomidine by continuous infusion for more than 1 hour were included in the study. Data for this study were collected using a computerised system that automatically collects information on patients receiving dexmedetomidine. Electronic health records (EHRs) and clinical notes were reviewed to identify demographics, dose, duration of treatment, additional sedative/analgesic administration and any ADRs, including hypotension, hypertension, bradycardia and apnea were obtained. For the assessment of ADRs, hypotension was defined as systolic and diastolic blood pressure below the 10th percentile for postmenstrual age and lasting more than 1 hour.^19^ While hypotension is more commonly reported, hypertension may also occur, particularly in association with bolus administration.^20^ Hypertension was defined as a systolic blood pressure value above the 90th percentile for postmenstrual age. Bradycardia was defined as a heart rate of less than 90 beats/min,^21^ lasting more than 1 hour. The duration of dexmedetomidine administration was calculated from the documented start and stop times recorded in the EHR. Changes in the duration and dosage of the drug over the years were analysed. The relationship between ADRs and factors such as prematurity or drug dose was examined. Cases of dose reduction or discontinuation due to ADRs lasting for more than 1 hour were also recorded.

**Figure 1:**
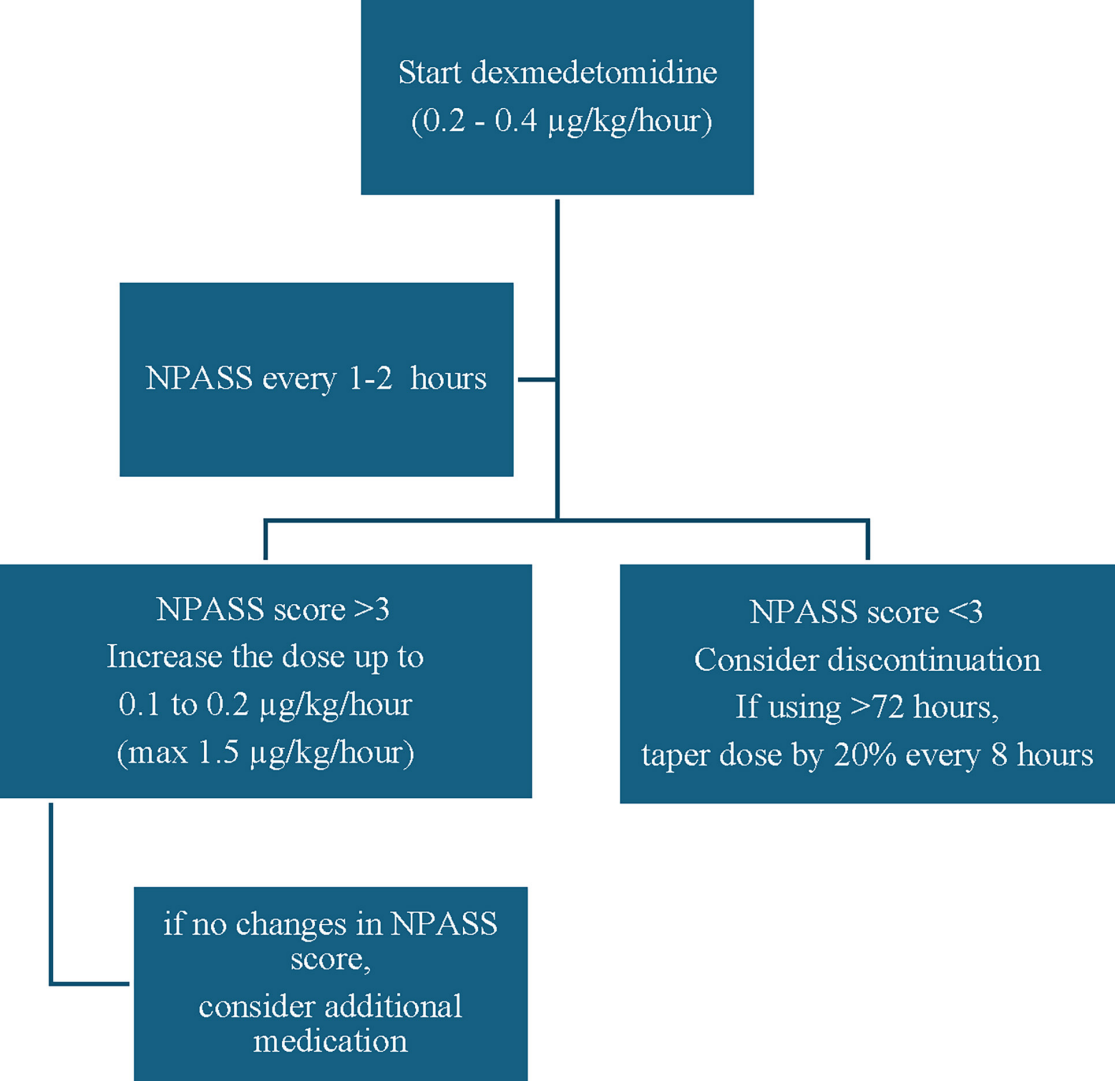
Dexmedetomidine dosing protocol. NPASS, Neonatal Pain, Agitation and Sedation Scale.

During the NICU hospitalisation, pain assessment was conducted using the Neonatal Pain, Agitation and Sedation Scale (NPASS). Patients with an NPASS score above 3 received appropriate pain-relieving interventions. Non-pharmacological and pharmacological treatments were selected according to the underlying condition. NPASS assessments were performed either hourly or every 2 hours throughout the duration of dexmedetomidine therapy for each patient. In our dexmedetomidine protocol, the drug was started at 0.2–0.4 µg/kg/hour and titrated in 0.1–0.2 µg/kg/hour increments based on pain and sedation assessment scores (figure 1). If it was used for more than 72 hours, it was tapered by 20% every 8 hours during discontinuation. Withdrawal symptoms, including tachycardia, agitation and hypertension, were monitored in this study due to the absence of a validated alpha-2 agonist withdrawal assessment tool. In the assessment of withdrawal symptoms, tachycardia was defined as a heart rate greater than 160 beats/min.

Demographic data, medication characteristics and ADRs profile were compared between term and preterm newborns. To investigate the relation between dosage and ADRs, the patients were divided into two groups based on the maximum dose received (either lower or higher than 0.5 µg/kg/hour) and these two groups were compared with each other. The threshold of 0.5 µg/kg/hour was chosen based on the published threshold by Dersch-Mills *et al*.^18^ Additionally, logistic regression, univariate and multivariate analyses were performed to identify factors influencing the occurrence of ADRs.

### Statistical analysis

Descriptive statistics were used to summarise patient demographics, indications for dexmedetomidine use, treatment duration, dosage and ADRs. Continuous variables are presented as mean (SD), while categorical variables are presented as frequencies and percentages. Statistical analyses were performed using SPSS V.26.0 (IBM Corp, Armonk, NY, USA). Two independent groups with an ordinal outcome or continuous data with not a normal distribution were assessed using the Mann-Whitney U test. P values <0.05 were considered statistically significant. χ^2^ test was used for two independent groups with nominal data. Pearson correlation was used to investigate the correlation between the mean initial or maximum dose and the years. Logistic regression, univariate and multivariate analyses were performed to investigate the effect of variables on ADRs, respectively.

## Results

### Demographic profiles

**Figure 2:**
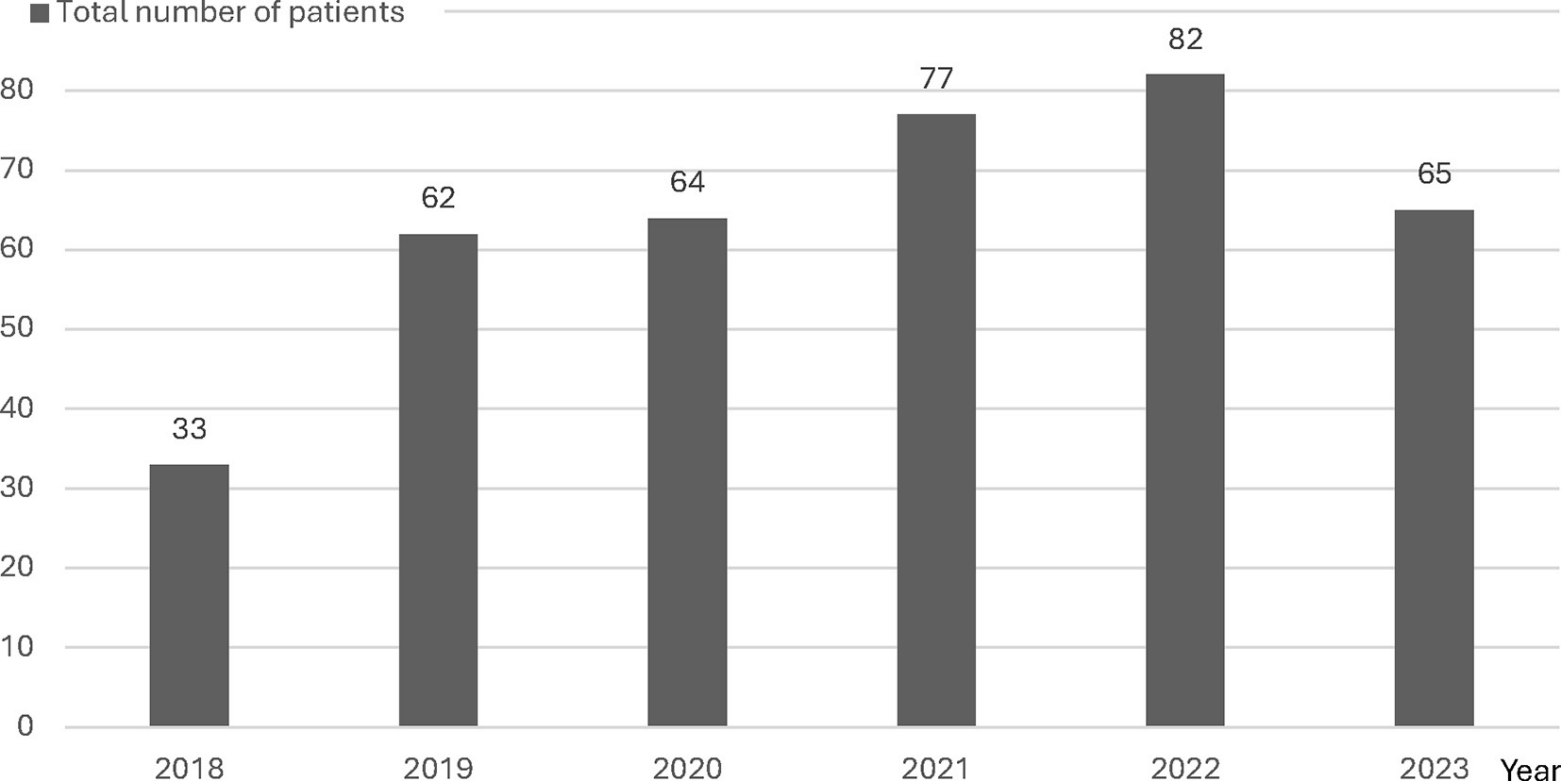
Dexmedetomidine administration trends over time.

**Figure 3:**
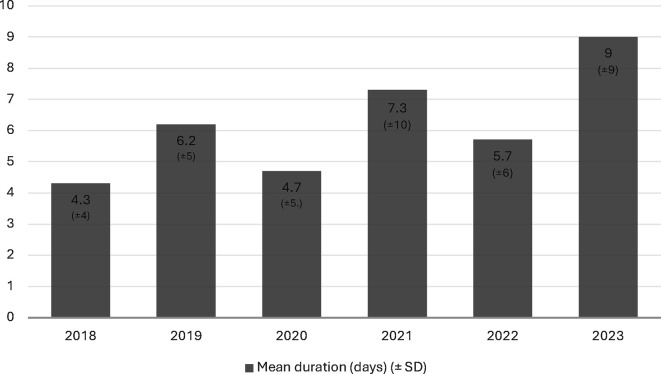
Trends in the mean duration time (days) of dexmedetomidine over years

A total of 4107 infants were admitted to the NICU between 2018 and 2023. A total of 383 infants (9%) received dexmedetomidine during their NICU stay, of which 140 (36.5%) were preterm. The number of patients who received dexmedetomidine by year is shown in figure 2. Demographic data, including gestational age, birth weight and gender, are presented in table 1. The mean (SD) postmenstrual age at the time of dexmedetomidine initiation was 38 (0.2) weeks and the median (IQR) postnatal age at initiation was 9 (3–20) days. The median (IQR) duration of treatment was 3 (2–8) days. Figure 3 illustrates the evolution of the duration of drug use over the years in the NICU. The Pearson correlation showed a significant relationship (p=0.009, r=0.204). Medication characteristics based on term and preterm birth are shown in table 1.

**Table 1:** Medication characteristics based on term and preterm birth

	Total (n=383)	Preterm (n=140)	Term (n=243)	P value
Gestational age (weeks) median (IQR)	37 (35–38)	32 (28–36)	38 (37–39)	**<0.001** *
Birth weight (g) median (IQR)	2700 (2140–3270)	1732 (900–2471)	3100 (2700–3480)	**<0.001** *
Gender (male) frequency (%)	196 (51.2)	65 (46.4)	131 (53.9)	0.16†
Duration of drug administration (days) mean (SD)	6.3 (0.4)	6.6 (7.4)	6.2 (6.8)	0.44*
Initial dose (µg/kg/hour) mean (SD)	0.3 (0.1)	0.32 (0.3)	0.29 (0.2)	0.95*
Maximum dose (µg/kg/hour) mean (SD)	0.45 (0.2)	0.48 (0.59)	0.43 (0.26)	0.44*
Postnatal age at initiation (days), median (IQR)	9 (3-20)	14 (3–30)	8 (3-15)	**0.001** *
Postmenstrual age at initiation (weeks), median (IQR)	38 (36–40)	35 (31–37)	39 (38–41)	**<0.001** *

Boldface font indicates a statistically significant variable.

* Mann-Whitney U test was administered to compare differences between groups.

† χ2 test was administered to analyse the differences between groups.

**Figure 4:**
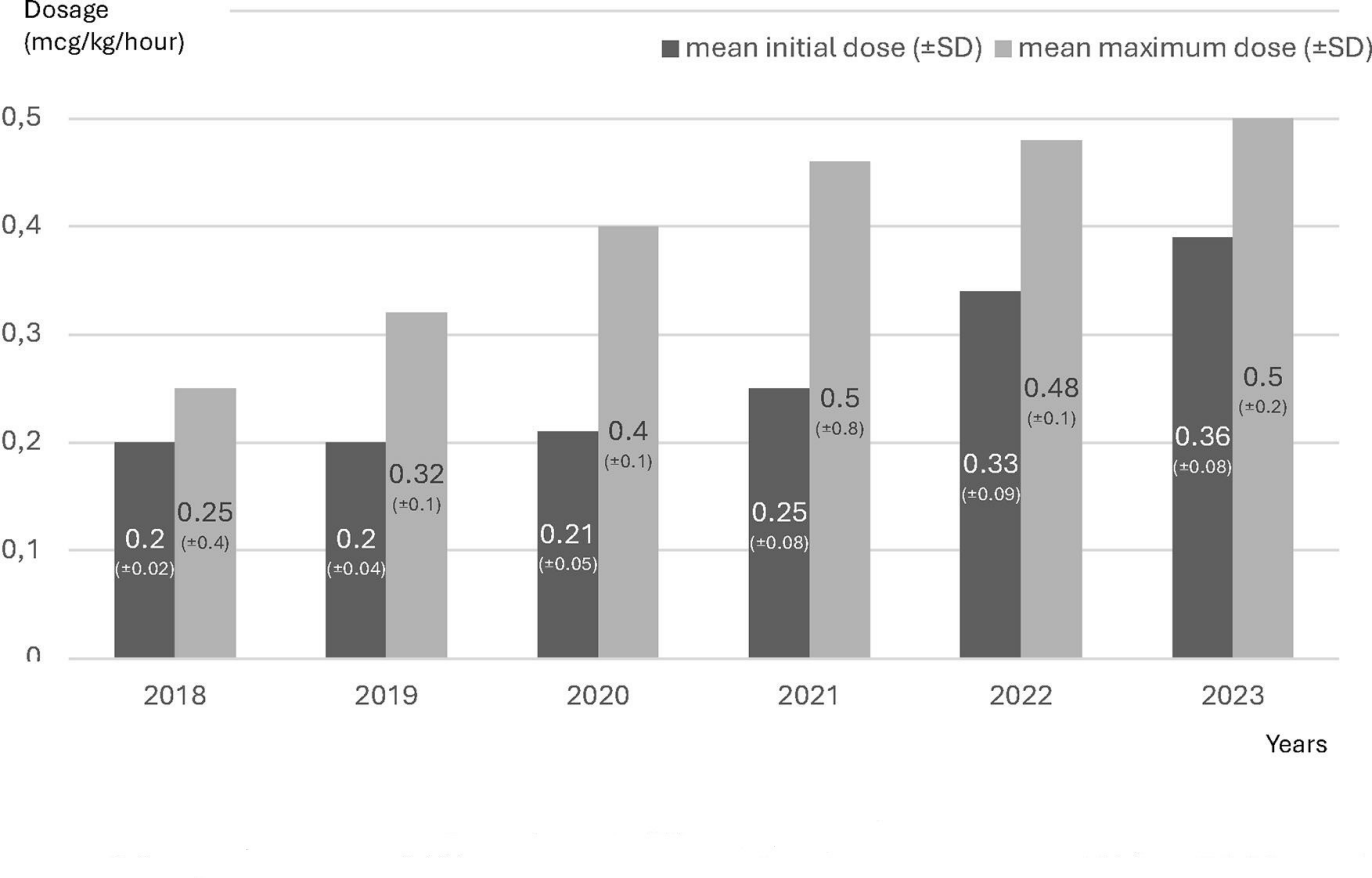
Dexmedetomidine mean initial and maximum dosage trends (µg/kg/hour) over years

The overall dosage range varied from 0.2 to 1.5 µg/kg/hour. The changes in dose over the years are shown in figure 4. The Pearson correlation test revealed a significant relationship (p<0.001, r=0.580 for the initial dose over the years; p=0.027, r=0.152 for the maximum dose over the years).

The most common indication for dexmedetomidine use was postoperative pain or pain following an interventional procedure (n=312, 81.5%). Of the total cohort, 351 (91.6%) patients underwent surgery. Additionally, dexmedetomidine was administered to 71 patients for management of pain or agitation not related to interventional procedures.

### ADR profiles

ADRs were observed in a total of 20 patients (5%). Overall, half of them (10 patients) required either dose reduction or discontinuation of the drug due to ADRs. Among ADRs, bradycardia was the most frequently reported one, affecting 10 patients. As a result, the drug was discontinued in three patients, while the dose had to be reduced in three patients. Hypotension was the second most common ADR, occurring in eight patients, with dose reduction necessary in two patients and discontinued in one case. Additionally, apnea was reported in two patients, leading to discontinuation in one case. There were no incidents of hypertension. The incidence of these ADRs in preterm and term infants was displayed in table 2. There was also no significant difference in terms of ADRs for patients who received dexmedetomidine before 37 weeks PMA versus 37 weeks PMA or greater (p=0.17). Notably, a total of two patients who experienced apnoea were under 37 weeks PMA. The relationship between the ADR profile and the maximum drug dose administered was also examined; however, no significant difference was observed (tables2  3). Notably, there were no documented cases of withdrawal symptoms associated with the use of dexmedetomidine.

**Table 2:** Comparison of ADR profile with gestational age

	Preterm(n=140)	Term(n=243)	P value
Bradycardia, n (%)	4 (3%)	6 (2%)	0.61
Hypotension, n (%)	4 (3%)	4 (2%)	0.39
Any interventions due to ADRs,*n (%)	3 (2%)	7 (3%)	0.67

* Interventions involved reducing the dose or discontinuing the drug in response to ADRs. χ2 test was administered to analyse the differences between groups.

ADRs, adverse drug reactions.

**Table 3:** Comparison of ADR profile with highest drug doses

	Maximum dose<0.5 µg/kg/hour(n=255)	Maximum dose≥0.5 µg/kg/hour (n=128)	P value
Bradycardia, n (%)	5 (2%)	5 (4%)	0.26
Hypotension, n (%)	7 (3%)	1 (1%)	0.28
Any interventions due to ADRs,*n (%)	5 (2%)	5 (4%)	0.26

* Interventions involved reducing the dose or discontinuing the drug in response to ADRs. χ2 test was administered to analyse the differences between groups.

ADRs, adverse drug reactions.

A multivariate analysis of factors influencing the occurrence of any ADRs is shown in table 4. The model included variables such as gestational age, maximum doses, initial chronological age, other concomitant analgesics, vasopressor support, duration of the treatment and cumulative dose.

**Table 4:** Multivariate analysis of factors influencing the occurrence of ADRs

	Univariate		Multivariate*	
	OR (95% Cl)	P value	aOR	P value
Gestational age	0.934 (0.843–1.033)	0.184	0.901 (0.801–1.015)	0.08
Maximum dose	0.806 (0.858–1.127)	0.654	1.053 (0.880–1.259)	0.57
Chronological age at initiation	0.999 (0.976–1.023)	0.921	0.990 (0.961–1.021)	0.53
Concomitant other analgesics	1.812 (0.699–4.699)	0.221	2.975 (1.004–8.816)	**0.049**
Vasopressor support	1.078 (0.374–3.103)	0.890	0.881 (0.276–2.809)	0.83
Duration of the treatment	0.967 (0.890–1.051)	0.433	1.035 (0.887–1.208)	0.66
Cumulative dose	0.992 (0.975–1.009)	0.327	0.979 (0.943–1.017)	0.27

Out of the 383 infants exposed to dexmedetomidine, 127 (59%) were undergoing invasive mechanical ventilation during the administration.

Bold values indicate statistically significant results (p < 0.05).

* Multivariate logistic regression models included all variables given in the rows.

ADRs, adverse drug reactions; aOR, adjusted OR.

Among the total participants, 226 infants (59%) received dexmedetomidine exclusively. 147 infants (38%) concurrently received a fentanyl infusion along with dexmedetomidine. Additionally, 7 (1.8%) infants received benzodiazepines (midazolam), 2 (0.5%) infants received ketamine, and 1 (0.3%) infant received paracetamol in conjunction with dexmedetomidine. Among the patients who experienced ADRs, 8 were exclusively on dexmedetomidine, while 11 received dexmedetomidine with other drugs (p=0.08). There was no statistically significant difference between the two groups.

Inotropes were used during dexmedetomidine administration in 101 (26.4%) patients. Of these, 56 patients were diagnosed with congenital heart diseases, and 17 patients with congenital diaphragmatic hernia. The remaining 28 patients included 5 with vein of Galen aneurysm, 4 with gastrointestinal anomalies and others with conditions such as necrotising enterocolitis, infection or pneumothorax. Among these, dopamine was concomitantly administered in 70 (18.3%) patients, milrinone in 59 (15.4%) patients, epinephrine in 37 (9.7%) patients and dobutamine in 10 (2.6%) patients. All patients who received inotropes were in the postoperative period, and their inotropes were initiated prior to the administration of dexmedetomidine.

## Discussion

In this study, we analysed 383 neonates who were treated with dexmedetomidine. There was a noticeable uptrend in doses of dexmedetomidine usage in the NICU over the study period. Importantly, we established an ADR profile, with the incidence of ADRs being 5% and the frequency of dose adjustments or reductions due to ADRs at 3%. Concomitant other analgesics were identified as the sole factor influencing the occurrence of ADRs.

Dexmedetomidine has exhibited a lower ADR profile compared with standard care, as supported by studies such as O’Mara *et al*,^8^ where patients in the dexmedetomidine group required less additional sedation and experienced shorter durations of mechanical ventilation compared with those receiving fentanyl. Additionally, the use of dexmedetomidine was associated with accelerated meconium passage and the achievement of full enteral nutrition. These findings led to the increased use of dexmedetomidine in our unit, particularly among surgical patients, who accounted for 91% of the cohort. In a study including postoperative neonates, those receiving dexmedetomidine (mean (±SD) dose: 0.36 (±0.12) µg/kg/hour) were compared with those receiving standard care, and no significant differences were observed in episodes of hypotension or respiratory depression between groups.^22^ Nevertheless, they reported a higher incidence of bradycardia in the dexmedetomidine group (12.8% vs 5.1%). In our study, the incidence of bradycardia was markedly lower at 3% than their report, even though their bradycardia cut-off was slightly lower than ours (80 beats/min vs 90 beats/min). The higher incidence of bradycardia observed in their study may have been influenced by the greater use of opioids. In that study, 95% of the dexmedetomidine group received an opioid infusion, compared with 37.4% in our study. Notably, they reported that all subjects who experienced episodes of bradycardia in the referenced study were receiving concomitant opioid infusions at the time of their event. Furthermore, our findings also showed a significant relationship between ADRs and concomitant drugs in multivariate analysis.

Hypotension was found to be the second most common ADR in our study. In a study conducted with 38 infants who received dexmedetomidine, mostly surgical patients similar to our study, overall rates of hypotension were reported at 41.7%.^18^ They used a median maximum dose of 0.5 µg/kg/hour, similar to our study. However, despite the higher incidence of ADRs observed in their cohort compared with ours, they did not report any instances of drug discontinuation due to ADRs. Additionally, the hypotension definition was similar across the studies. They stated that the frequency of hypotension might be related to the postoperative period. Furthermore, O’Mara *et al*^8^ did not include any neonate requiring surgery and reported no significant effect of dexmedetomidine on blood pressure. Although these results seem to support the effect of the postoperative period on hypotension, our findings were not supported by it. In our study, although hypotension was observed in some cases, its incidence was relatively low compared with other studies that included postoperative patients. The observation of a higher incidence of hypotension in the postoperative period in the other studies may be related to the timing of the initiation of dexmedetomidine. Administering dexmedetomidine immediately after surgery, while the effects of anaesthesia are still present, could increase the risk of hypotension. However, details of the timing of dexmedetomidine initiation in previous studies have not been reported. In our study, we initiated the drug as the NPASS score increased, indicating that the effects of anaesthetic drugs were wearing off. This approach probably minimised the overlap between anaesthetic drugs and dexmedetomidine effects, resulting in fewer cases of hypotension.

In our study, we did not find any relationship between ADR profiles and dosages, or gestational age, based on both univariate and multivariate analysis. A study completed by Guillen-Hernandez *et al*^23^ reported on 104 preterm infants with a median gestational age of 26 weeks which is lower than our study population. The dose and duration were similar to our findings. They found the incidence of bradycardia was notably higher in neonates with a birth weight of less than 1000 g; however, no discontinuations of dexmedetomidine due to bradycardia were reported. They suggested that the increased incidence of bradycardia might be related to the infusion rate of dexmedetomidine, especially in infants weighing less than 1000 g, who required higher doses to achieve adequate comfort levels. Additionally, Estkowski *et al*^20^ found that bradycardia was more common in infants than in neonates and thought that this might be due to the use of higher doses in infants. While these studies suggest that the frequency of side effects may increase with higher doses, our findings do not support this correlation.

The use of dexmedetomidine has been increasing over time. In a multicentre study conducted by Curtis *et al*^13^ encompassing NICUs across the USA, a rise in dexmedetomidine utilisation since 2010 was demonstrated, coinciding with a decrease in opioid use. In our study, differences in dexmedetomidine utilisation over the years were observed over time. Interestingly, we have found a significant increase in both initial and maximum doses of dexmedetomidine administered with a significant increase in the duration of dexmedetomidine use over the years. This trend appears to be due to accumulated experience with its use and may also possibly reflect growing confidence in the safety profile of the drug. It may also indicate that the initial dose was not sufficient and that higher doses were needed to achieve the desired effect. This observation suggests that we may need to reconsider its efficacy compared with other drugs. Additionally, a meta-analysis showing that there are no randomised controlled trials on the use of dexmedetomidine in neonates suggests that the exact effectiveness of dexmedetomidine remains unclear.^24^ Therefore, it is clear that more randomised controlled studies are needed on this subject.

The strength of our study is highlighted by the large sample size, but it is important to acknowledge several limitations. First, our study did not directly evaluate efficacy, and was unable to compare the effects of dexmedetomidine with other medications and other groups. Second, it is difficult to determine whether ADRs such as hypotension or bradycardia were specific to dexmedetomidine or to other agents used in concomitant therapy. Third, our study primarily included surgical patients in a single centre and may not comprehensively capture ADRs in all populations. Additionally, the retrospective nature of the study, which relied on medical records, may have led to an underestimation of ADRs. Furthermore, as we were unable to measure dexmedetomidine plasma levels, dose adjustments were based on NPASS scores alone, so we were unable to confirm whether the drug was reaching therapeutic levels or to account for individual variations. Finally, the lack of long-term follow-up in our study limits our ability to assess outcomes beyond the neonatal period.

## Conclusion

To conclude, our study highlights an increase in dose and duration of dexmedetomidine in our NICU while the ADR profile has not been shown a correlation with dose and duration.

## Data Availability

Data are available upon reasonable request.
