# Supplementary material for: Dexmedetomidine in neonates: utilisation trends and safety profile over time in a neonatal intensive care unit
Source: BMJ Paediatr Open. 2025 Mar 18;9(1):e003004. doi: 10.1136/bmjpo-2024-003004 (PMC11927418; doi:10.1136/bmjpo-2024-003004)
Supplement: Supplementary file 1 [file bmjpo-2024-003004.reviewer_comments.pdf]

PEER REVIEW HISTORY

BMJ Paediatrics Open publishes all reviews undertaken for accepted manuscripts. Reviewers are asked to complete a checklist review form and are provided with free text boxes to elaborate on their assessment. These free text comments are reproduced below.

ARTICLE DETAILS

**TITLE (PROVISIONAL)**	Dexmedetomidine in Neonates: Utilization Trends and Safety Profile Over Time in a Neonatal Intensive Care Unit
**AUTHORS**	Kayki, Gozdem; Yalcin, Nadir; Celik, Tolga; Yigit, Sule

VERSION 1 - REVIEW

**REVIEWER NAME**	***Indrani Bhattacharjee***
**REVIEWER AFFILIATION**	Tufts University School of Medicine, Newborn Medicine
**REVIEWER CONFLICT OF INTEREST**	Not applicable
**DATE REVIEW RETURNED**	23-Sep-2024

**GENERAL COMMENTS**	Overall a very well done and well written study. The only caveat in this study will be a lack of clear description of the units dosing protocol. The study shows an increasing trend in both the dose and duration of dexmedetomidine administration over time. However, there is no standardized dosing protocol across the study period, which may suggest that initial doses were insufficient, and clinicians gradually increased them as they gained more experience. Can the authors elaborate of the dosing protocol and hence emphasize the external validity of the study findings beyond the study NICU

**REVIEWER NAME**	***Saminathan Anbalagan***
**REVIEWER AFFILIATION**	University of South Alabama
**REVIEWER CONFLICT OF INTEREST**	Not applicable
**DATE REVIEW RETURNED**	02-Oct-2024

**GENERAL COMMENTS**	This study examines the trends in the use of dexmedetomidine in a single level 4 referral neonatal intensive care unit (NICU) (in Turkey?) and the associated adverse drug reactions (ADRs). The authors conclude that the proportion of patients on dexmedetomidine had increased in their NICU, and there was a " significant increase in the dose and duration of dexmedetomidine use" without an increase in ADRs at "higher doses" or "at any gestational age." However, the results of this study are overstated, oversimplified, and have methodological flaws that hinder appropriate conclusions.<br><br>Major:<br>-The abstract is missing the country of study, statistical methods used, basic demographics of the patients, and p-value for the

	statistically significant findings. The conclusion is strongly worded with misleading claims that the drug is safe at "higher doses" and at "any gestational age."<br>-The study is not comprehensive about ADRs and mainly included surgical patients.<br>-The methods section lacks details such as the place of study (city and country where the NICU is situated) and validated reasons for choosing the cutoff values and duration for each ADR. Choosing a clinically meaningful threshold using validated scales is necessary.<br>-The statistical methods used do not seem appropriate for the stated objectives of this study. ANOVA was inappropriately used to compare the dose/duration of the drug across years. More appropriate tests that could be used are Pearson's correlation or linear mixed model. The authors must also clarify why a paired t-test was used.<br>-The specific statistical test employed for each reported result is missing.<br>-Since the reported ADR rate is very low, the McNemar test with Benjamini–Hochberg correction is a better statistical test.<br>-The denominator for interventions for ADRs (reducing the dose or discontinuing the drug) must be the total number of patients that had ADRs and not the entire cohort. Hence, 50% (10 out of 20) of these patients needed some form of intervention for the ADR, and this highly contradicts the stated results. This, by itself, raises significant concern about the safety profile of the drug. Moreover, the authors draw a strong causal relation to the findings, but this is not a randomized controlled trial to report such conclusions.<br>-The authors did not include important confounding variables in the reported multivariate logistic regression model, such as the drug duration and cumulative dose, as these have also been associated with the reported ADRs.<br>-Authors report collection of withdrawal symptoms, but such data is not presented.<br>-Incidence of other ADRs, such as hypertension, is not reported.<br>-The authors compare the ADRs between the "maximum dose" in Table 2 - the reasoning for the decision to split the maximum dose at 0.5 mcg/kg/hr is lacking.<br>-Did the ADR occur in the same patient repeatedly? If so, it must be mentioned and reported.<br>-The discussion section is rather all over the place without a coherent correlation between this study's findings and the literature. It is too long and biased towards studies that reported safety. Authors have repeatedly referred to the results of other studies, but the variables from those studies were not examined in this study and hence irrelevant.<br>-The study's limitations must also include a lack of generalizability due to a single-center study, lack of comparison group, and inability to demonstrate causation as it is a retrospective observational study.<br>-The authors draw firm conclusions, such as "established low ADR" and "no correlation with gestational age or dose," from methodologically flawed results. Moreover, they must stress the need for further research with RCTs to identify the safety of this drug in neonates rather than claiming it is already safe to use.<br><br>Minor:<br>-The title page is missing the authors' degree and title.

	-Claims such as "the first study to..." or "first time in research" are inaccurate and must be avoided.<br>-All three paragraphs of the introduction are lengthy and lack specificity for the reasons behind doing this study.<br>-Paragraph 3 of the introduction discusses why dexmedetomidine is beneficial, but it is more suited to the discussion section rather than the introduction.<br>-Abbreviations such as NPASS are not expanded in the first instance (page 5, line 52).<br>-There are multiple awkward phrasing across the manuscript, such as "means of initial dose and maximum dose in years," "general dose range," or "reported one," and a mix of active and passive tenses (a reference to Table 2 on page 8, line 30). It is suggested that an English grammar tool be used in the future.<br>-Table 1 lacks baseline patient characteristics like gestational age, birth weight, and gender for each group.<br>-Table 2 is not represented in a standard format. I suggest splitting it and having ADRs as rows.<br>-Across the manuscript, all statistically significant p-values <0.05 must be expanded to the actual value obtained and up to 3 decimal points. p-value is not needed if the findings are not statistically significant.<br>-Results are repeated in the text and the tables.<br>-New results are introduced for the first time in the discussion section.<br>-Legends are missing for Tables & Figures, including the statistical test(s) used.<br>-Figures don't have measures of intervals such as SD.

VERSION 1 – AUTHOR RESPONSE

Reviewer: 1

Comments to the Author

Overall a very well done and well written study. The only caveat in this study will be a lack of clear description of the units dosing protocol. The study shows an increasing trend in both the dose and duration of dexmedetomidine administration over time. However, there is no standardized dosing protocol across the study period, which may suggest that initial doses were insufficient, and clinicians gradually increased them as they gained more experience. Can the authors elaborate of the dosing protocol and hence emphasize the external validity of the study findings beyond the study NICU

- Author response: We appreciate your valuable feedback. We have addressed your comment by elaborating on the dosing protocol in the revised manuscript, now presented as Figure 1. Specifically, our protocol outlines an initial dosing range of 0.2–0.4 µg/kg/hour, with clinicians generally starting at the lower end of this range. Over time, the trend observed in our study indicates a gradual shift towards the use of the upper limit of the range. This progression likely reflects increasing clinical experience and confidence in using dexmedetomidine in this population. Moreover, this finding suggests that starting at the upper limit of the dosing range may provide more effective results for certain patient groups. We believe this context enhances the external validity of our findings and will provide valuable insights for other NICUs considering similar protocols.

Reviewer: 2

Comments to the Author

This study examines the trends in the use of dexmedetomidine in a single level 4 referral neonatal intensive care unit (NICU) (in Turkey?) and the associated adverse drug reactions (ADRs). The authors conclude that the proportion of patients on dexmedetomidine had increased in their NICU, and there was a "significant increase in the dose and duration of dexmedetomidine use" without an increase in ADRs at "higher doses" or "at any gestational age." However, the results of this study are overstated, oversimplified, and have methodological flaws that hinder appropriate conclusions.

Major:

-The abstract is missing the country of study, statistical methods used, basic demographics of the patients, and p-value for the statistically significant findings. The conclusion is strongly worded with misleading claims that the drug is safe at "higher doses" and at "any gestational age."

-- Author response: Thank you for your valuable feedback. We have revised the abstract to include the country where the study was conducted, the statistical methods used, and the basic demographics of the patients. Additionally, we have provided p-values for the statistically significant findings. The conclusion has also been adjusted to avoid strong claims.

-The study is not comprehensive about ADRs and mainly included surgical patients.

-- Author response: Thank you for your observation. We acknowledge that our study primarily included surgical patients, as this reflects the predominant patient demographic managed by our unit. While this focus may limit the comprehensiveness of ADRs in non-surgical populations, it offers valuable insights specific to this high-risk cohort. To address your concerns, we have explicitly included this point as a limitation in the revised manuscript to provide appropriate context for the study findings.

“Thirdly, our study primarily included surgical patients in a single center and may not comprehensively capture ADRs in all populations.” in last paragraph on Page 13.

-The methods section lacks details such as the place of study (city and country where the NICU is situated) and validated reasons for choosing the cutoff values and duration for each ADR. Choosing a clinically meaningful threshold using validated scales is necessary.

-- Author response: We have added the city and country where the NICU is located to the revised methods section. Regarding the cutoff values, we based our approach on the definition of the neonatal period. For the duration, we selected a threshold of more than one hour to ensure that the observed changes were not due to transient fluctuations. These clarifications have been incorporated into the revised manuscript.

Cutoff values for bradycardia: “Killen SAS, Fish FA. Fetal and Neonatal Arrhythmias. NeoReviews 2008; 9(6): e242-e252.

Cutoff values for hypotension: “Sahni M, Jain S. Hypotension in Neonates. NeoReviews 2016; 17(10): e579-e589.

-The statistical methods used do not seem appropriate for the stated objectives of this study. ANOVA was inappropriately used to compare the dose/duration of the drug across years. More appropriate tests that could be used are Pearson's correlation or linear mixed model. The authors must also clarify why a paired t-test was used.

-- Author response: Thank you for your feedback regarding the statistical methods. A Pearson's correlation analysis was conducted, revealing a significant correlation between years and the initial dose ($r = .204$, $n = 383$, $p < .001$). The statistical analysis section has been revised and written in detail. The statistical analysis section has been revised and now includes a detailed explanation of the methods used. Additionally, we have removed any reference to a paired t-test, as it was not used in the final analysis. The updated statistical analysis can be found on page 6 of the revised manuscript.

-The specific statistical test employed for each reported result is missing.

-- Author response: We have now added the specific statistical tests employed for each reported result in the revised manuscript.

-Since the reported ADR rate is very low, the McNemar test with Benjamini–Hochberg correction is a better statistical test.

-- Author response: Thank you for your suggestion. In our study, the reported ADR rates were compared between preterm and term groups, as well as between lower and higher doses, using the Chi-square test. The McNemar test is typically used for paired groups, but since our groups are not paired, we believe the Chi-square test is the more appropriate statistical method in this context.

-The denominator for interventions for ADRs (reducing the dose or discontinuing the drug) must be the total number of patients that had ADRs and not the entire cohort. Hence, 50% (10 out of 20) of these patients needed some form of intervention for the ADR, and this highly contradicts the stated results. This, by itself, raises significant concern about the safety profile of the drug. Moreover, the authors draw a strong causal relation to the findings, but this is not a randomized controlled trial to report such conclusions.

-- Author response: We would like to clarify that our study is retrospective in nature, and we are presenting the findings as observed. Our intention in reporting the data in this way was to provide an overview of how many patients required dose reduction or discontinuation of the drug due to ADRs. To address this concern, we have revised the result section for clarity: "ADRs were observed in a total of 20 patients (5%). Overall, half of them (10 patients) required either dose reduction or discontinuation of the drug due to ADRs." On the 8th page (2nd paragraph).

-The authors did not include important confounding variables in the reported multivariate logistic regression model, such as the drug duration and cumulative dose, as these have also been associated with the reported ADRs.

-- Author response: Thank you for your comment. We have now included both drug duration and cumulative dose in the multivariate logistic regression model. Please refer to Table 4 in the revised manuscript for the updated analysis.

-Authors report collection of withdrawal symptoms, but such data is not presented.

-- Author response: As mentioned in the results section in the last paragraph on the 8th page, we did not observe withdrawal symptoms in our study cohort.

-Incidence of other ADRs, such as hypertension, is not reported.

-- Author response: There were no observations of hypertension as an ADR in our study.

-The authors compare the ADRs between the "maximum dose" in Table 2 - the reasoning for the decision to split the maximum dose at 0.5 mcg/kg/hr is lacking.

-- Author response: The decision to split the "maximum dose" at 0.5 µg/kg/hr was based on the established threshold, as described by Dersch-Mills, which categorizes doses below 0.5 µg/kg/hr as low and those above as high. We added this information in Methods section (the second paragraph on the 6th page).

“To investigate the relation between dosage and ADRS, the patients were divided into two based on the maximum dose received (either lower or higher than 0.5 µg/kg/hr) and these two groups were compared with each other. The threshold of 0.5 µg/kg/hr was chosen based on the published threshold by Dersch-Mills et al.10”

*Dersch-Mills DA, Banasch, HL, Yusuf K, Howlett A. Dexmedetomidine Use in a Tertiary Care NICU: A Descriptive Study. Ann Pharmacother. 2019;53(5):464–470.

-Did the ADR occur in the same patient repeatedly? If so, it must be mentioned and reported.

-- Author response: The ADRs did not occur repeatedly in the same patient; each ADR was observed in a different patient.

-The discussion section is rather all over the place without a coherent correlation between this study's findings and the literature. It is too long and biased towards studies that reported safety. Authors have repeatedly referred to the results of other studies, but the variables from those studies were not examined in this study and hence irrelevant.

-- Author response: We have thoroughly revised the discussion section to improve its coherence and organization. The first paragraph provides a general summary of the study. The second discusses bradycardia as a side effect, exploring potential reasons for its lower frequency in our study. The third focuses on hypotension-related side effects. The fourth paragraph addresses publications on side effects and factors that may influence them. The fifth examines the frequency of use and dosage. Finally, the last paragraph highlights the study’s limitations. We hope these revisions have resulted in a more structured and concise discussion section.

-The study's limitations must also include a lack of generalizability due to a single-center study, lack of comparison group, and inability to demonstrate causation as it is a retrospective observational study.

-- Author response: Thank you for your comment. We have now included additional limitations in the revised manuscript.

-The authors draw firm conclusions, such as "established low ADR" and "no correlation with gestational age or dose," from methodologically flawed results. Moreover, they must stress the need for further

research with RCTs to identify the safety of this drug in neonates rather than claiming it is already safe to use.

-- Author response: Thank you for your comment. We acknowledge the importance of conducting more randomized controlled trials to better establish the safety and efficacy profile of this drug in neonates. This point has been emphasized on page 13. Furthermore, we have revised our conclusions to reflect a more cautious interpretation of our findings, specifying that no associations were observed in our study rather than making definitive claims.

Minor:

-The title page is missing the authors' degree and title.

-- Author response: We have added the authors' degrees and titles to the title page in the revised manuscript.

-Claims such as "the first study to..." or "first time in research" are inaccurate and must be avoided.:

-- Author response: We have corrected the manuscript to avoid claims such as "the first study to..." or "first time in research" and revised the text accordingly.

-All three paragraphs of the introduction are lengthy and lack specificity for the reasons behind doing this study.

-- Author response: We have revised the introduction to make it more concise and focused.

-Paragraph 3 of the introduction discusses why dexmedetomidine is beneficial, but it is more suited to the discussion section rather than the introduction.

-- Author response: Thank you for your comment. We have revised Paragraph 3 of the introduction to make it more concise and focused on the study's context.

-Abbreviations such as NPASS are not expanded in the first instance (page 5, line 52).

-- Author response: We have corrected this by expanding abbreviations of NPASS at their first mention in the manuscript.

-There are multiple awkward phrasing across the manuscript, such as "means of initial dose and maximum dose in years," "general dose range," or "reported one," and a mix of active and passive tenses (a reference to Table 2 on page 8, line 30). It is suggested that an English grammar tool be used in the future.

-- Author response: We have revised the text to improve clarity and consistency. Specifically, we have corrected the phrasing you mentioned. Additionally, we have made revisions to Table 2 to enhance its clarity.

“was used to investigate correlation between the mean initial or maximum dose and the years”

“The overall dosage range varied from 0.2 and 1.5 mcg/kg/hour.”

-Table 1 lacks baseline patient characteristics like gestational age, birth weight, and gender for each group.

-- Author response: We have added baseline patient characteristics, including gestational age, birth weight, and gender for each group, to Table 1 in the revised manuscript

-Table 2 is not represented in a standard format. I suggest splitting it and having ADRs as rows.

-- Author response: Thank you for your suggestion. We have split the original Table 2 into two tables: Table 2 and Table 3. In the revised format, ADRs are presented as rows in Tables.

-Across the manuscript, all statistically significant p-values <0.05 must be expanded to the actual value obtained and up to 3 decimal points. p-value is not needed if the findings are not statistically significant.

-- Author response: Thank you for your observation. We have revised the manuscript to include actual p-values up to three decimal points for all statistically significant findings.

-Results are repeated in the text and the tables.

-- Author response: We have revised the manuscript to reduce repetition by ensuring that results are presented either in the text or in the tables, but not both.

-New results are introduced for the first time in the discussion section.

-- Author response: We have thoroughly reviewed the discussion section and confirmed that no new results are introduced. All results mentioned in the discussion are based on findings already presented in the results section.

-Legends are missing for Tables & Figures, including the statistical test(s) used.

-- Author response: We have added legends for all Tables and Figures, including the statistical tests used.

-Figures don't have measures of intervals such as SD.

-- Author response: We have added standard deviations to the figures in the revised manuscript.

VERSION 2 – REVIEW

**REVIEWER NAME**	***Indrani Bhattacharjee***
**REVIEWER AFFILIATION**	Tufts University School of Medicine, Newborn Medicine
**REVIEWER CONFLICT OF INTEREST**	N/A
**DATE REVIEW RETURNED**	28-Dec-2024

**GENERAL COMMENTS**	no other changes meeded, thankyou

**REVIEWER NAME**	***Saminathan Anbalagan***
**REVIEWER AFFILIATION**	University of South Alabama
**REVIEWER CONFLICT OF INTEREST**	N/A
**DATE REVIEW RETURNED**	31-Dec-2024

**GENERAL COMMENTS**	Thank you to the authors for making the requested changes. The updated manuscript flows better and is more appropriate now. Minor changes are suggested below.<br><br>- Authors report only details about hypotension and bradycardia as the ADRs. However, throughout the manuscript, they claim that the "ADR profile" is safe with higher doses/duration. There have been reports of hypertension with higher infusion rates and also some cases of respiratory depression in non-invasively ventilated infants. If these ADRs were not collected, it must be stated explicitly in the limitations section, and authors must refrain from using the term "ADR profile" throughout, as they have collected data only on hypotension and bradycardia, which is not comprehensive. Or authors may consider rewording to "cardiovascular ADRs" throughout the manuscript.<br>- Thank you for providing the references for the cutoff value for bradycardia and hypotension in the response letter. Please include those references in the manuscript.<br>- In logistic regression, please describe if a specific ADR or occurrence of any ADR was chosen as the dependent variable.<br>- The result of the Pearson correlation test needs to be stated in the results section, including the r value. It is not enough to mention the p-value within the figure.<br>- It is suggested that gestational age should be represented by median and IQR (with appropriate statistical tests), as values like "3.9 weeks" are not meaningful for readers.<br>- Table 4: birth weight is not reported in the regression analysis when it was statistically different between preterm and term (authors claim in the discussion section it was included).<br>- Please correct the grammar on Page 10, line 47 "...identified as an only factor."<br>- Please rephrase/correct grammar on Page 11, line 40, "...involving 38 infants administered dexmedetomidine".<br>- Page 11, line 52: Authors need to clarify whether the differences are due to differences in definition or not. Currently, the manuscript states both.<br>- Page 12, line 10: "...observation of a higher incidence," but this study had a lower incidence. Please clarify.<br>- Page 12, line 42: "... birth weight of less than 1000g" - Authors continue to report new findings in the discussion section - please state it first in the results section!<br>- When reporting nonsignificant p-values, only two decimal values are needed (e.g., page 2, line 45 - p=0.934 should be 0.93). Please correct all such numbers throughout the manuscript.

VERSION 2 – AUTHOR RESPONSE

Reviewer #2:

- Authors report only details about hypotension and bradycardia as the ADRs. However, throughout the manuscript, they claim that the "ADR profile" is safe with higher doses/duration. There have been reports of hypertension with higher infusion rates and also some cases of respiratory depression in non-invasively ventilated infants. If these ADRs were not collected, it must be stated explicitly in the limitations section, and authors must refrain from using the term "ADR profile" throughout, as they have collected data only on hypotension and bradycardia, which is not comprehensive. Or authors may consider rewording to "cardiovascular ADRs" throughout the manuscript.

-Author response: Thank you for your comment. In addition to hypotension and bradycardia, we also observed and reported two cases of apnea. Hypertension was not detected in our study. We have revised the Methods section to provide more detailed information regarding the collection and reporting of ADRs on page 5.

- Thank you for providing the references for the cutoff value for bradycardia and hypotension in the response letter. Please include those references in the manuscript.

-Author response: We have added the references for the cutoff values for bradycardia and hypotension to the manuscript as requested.

- In logistic regression, please describe if a specific ADR or occurrence of any ADR was chosen as the dependent variable.

-Author response: Thank you for your question. In the logistic regression analysis, the occurrence of any ADR was chosen as the dependent variable.

- The result of the Pearson correlation test needs to be stated in the results section, including the r value. It is not enough to mention the p-value within the figure.

-Author response: Thank you for your comment. We have mentioned the r-value of the Pearson correlation test in the results section as requested.

- It is suggested that gestational age should be represented by median and IQR (with appropriate statistical tests), as values like "3.9 weeks" are not meaningful for readers.

-Author response: Thank you for your suggestion. We have updated the representation of gestational age to median and interquartile range (IQR) as recommended.

- Table 4: birth weight is not reported in the regression analysis when it was statistically different between preterm and term (authors claim in the discussion section it was included).

-Author response: Thank you for pointing this out. You are correct, and we have removed the mention of birth weight from the regression analysis to align with the data presented in Table 4.

- Please correct the grammar on Page 10, line 47 "...identified as an only factor."

-Author response: Thank you for your comment. We have corrected the grammar on Page 10, by changing the phrase to “as the sole".

- Please rephrase/correct grammar on Page 11, line 40, "...involving 38 infants administered dexmedetomidine".

-Author response: Thank you for your suggestion. We have corrected the phrase on Page 11, to “conducted with 38 infants who received dexmedetomidine.”

- Page 11, line 52: Authors need to clarify whether the differences are due to differences in definition or not. Currently, the manuscript states both.

-Author response: Thank you for your comment. We have clarified that there were no differences due to variations in definition, ensuring consistency in the manuscript.

- Page 12, line 10: "...observation of a higher incidence," but this study had a lower incidence. Please clarify.

-Author response: Thank you for your comment. We intended to emphasize the higher incidence observed in other studies. We have revised the sentence for clarity to reflect this.

- Page 12, line 42: "... birth weight of less than 1000g" - Authors continue to report new findings in the discussion section - please state it first in the results section!

-Author response: Thank you for your comment. We clarified that the statement regarding a birth weight of less than 1000g is not part of our findings. The sentence has been revised to prevent any misunderstanding.

- When reporting nonsignificant p-values, only two decimal values are needed (e.g., page 2, line 45 - p=0.934 should be 0.93). Please correct all such numbers throughout the manuscript.

-Author response: Thank you for your comment. We have corrected all nonsignificant p-values throughout the manuscript to show only two decimal places, as requested.

VERSION 3 - REVIEW

**REVIEWER NAME**	***Indrani Bhattacharjee***
**REVIEWER AFFILIATION**	Tufts University School of Medicine, Newborn Medicine
**REVIEWER CONFLICT OF INTEREST**	Not applicable
**DATE REVIEW RETURNED**	24-Jan-2025

**GENERAL COMMENTS**	No other revision recommended

**REVIEWER NAME**	***Saminathan Anbalagan***
**REVIEWER AFFILIATION**	University of South Alabama
**REVIEWER CONFLICT OF INTEREST**	Not applicable
**DATE REVIEW RETURNED**	07-Feb-2025

**GENERAL COMMENTS**	Thank you for making the suggested changes.<br><br>Please address the following as well:<br>- In the abstract, please use median (IQR) to represent post menstrual age at initiation, to have consistency with gestational age.<br>- Please including a sentence in the results section that there were no incidence of hypertension in this study.<br>- Table 1, abstract, and results section show Mann-Whitney U (MWU) test was used for birth weight but it is reported in mean and SD. Please report in median and IQR if the MWU test was used.<br>- Table 2: please correct "<0.616" to actual p value and with 2 decimals, because the current value can be interpreted as statistically significant.<br>- Table 4: please correct "p" to "p value".<br>- Also, please use lower case in all tables for p value.<br>- Table 4: concomitant other analgesics' p value is statistically significant, so please change it back to 0.049

VERSION 3 – AUTHOR RESPONSE

We sincerely appreciate the detailed and constructive feedback from the reviewers. Their insightful comments have helped improve the quality of my manuscript. We have carefully addressed all the suggested revisions in this revision, and we are grateful for the opportunity to refine our work.

Reviewer:

- In the abstract, please use median (IQR) to represent post menstrual age at initiation, to have consistency with gestational age.

Author response: Thank you. It has been revised.

- Please including a sentence in the results section that there were no incidence of hypertension in this study.

Author response: Thank you. We have added the sentence: 'There were no incidents of hypertension in this study.'

- Table 1, abstract, and results section show Mann-Whitney U (MWU) test was used for birth weight but it is reported in mean and SD. Please report in median and IQR if the MWU test was used.

Author response: Thank you. The correction has been made, and birth weight is now reported as median and IQR.

- Table 2: please correct "<0.616" to actual p value and with 2 decimals, because the current value can be interpreted as statistically significant.

Author response: The p-value has been corrected and now appears with two decimal places.

- Table 4: please correct "p" to "p value".

Author response: The correction has been made, and 'p' is now listed as 'p value'.

- Also, please use lower case in all tables for p value.

Author response: The correction has been made, and 'p value' is now in lowercase in all tables.

- Table 4: concomitant other analgesics' p value is statistically significant, so please change it back to 0.049-

Author response: The p-value for 'concomitant other analgesics' has been corrected to 0.049.
